# Substrate utilization and secondary metabolite biosynthesis in the phylum *Planctomycetota*

**DOI:** 10.1007/s00253-025-13514-1

**Published:** 2025-05-15

**Authors:** Madeleine Kündgen, Christian Jogler, Nicolai Kallscheuer

**Affiliations:** 1 Department of Microbial Interactions, Institute for Microbiology, Friedrich Schiller University, 07743 Jena, Germany; 2 Cluster of Excellence Balance of the Microverse, Friedrich Schiller University, 07745 Jena, Germany

**Keywords:** High-molecular weight sugars, Algae, Phototrophs, Aquatic bacteria, Secondary metabolites, Bioprospection, Bioactivity

## Abstract

**Abstract:**

The phylum *Planctomycetota* is changing our understanding of bacterial metabolism, driving critical biogeochemical processes through the transformation of complex polymeric substrates into valuable bioactive compounds. Sophisticated methods for cultivation, genome sequencing and genetic strain engineering developed in the last two decades have stimulated detailed studies on cell propagation, metabolic capabilities and potential applications of phylum members beyond the mere isolation and characterization of novel taxa. This review synthesizes recent advances in understanding the *Planctomycetota* physiology with a focus on the degradation of phototroph-derived polysaccharides, anaerobic ammonium oxidation (anammox) and biosynthesis of secondary metabolites. New data especially collected over the last 5 years justifies more intensive research of the yet uncharacterized pathways of substrate uptake and utilization, as well as genome mining-assisted bioprospection to exploit the phylum's chemical repertoire.

**Key points:**

• *Planctomycetes can degrade high-molecular-weight sugars produced by algae*

• *Anaerobic ammonium oxidation (anammox) is used in technical applications*

• *The first secondary metabolites were discovered in the last 5 years*

## Introduction

**Fig. 1:**
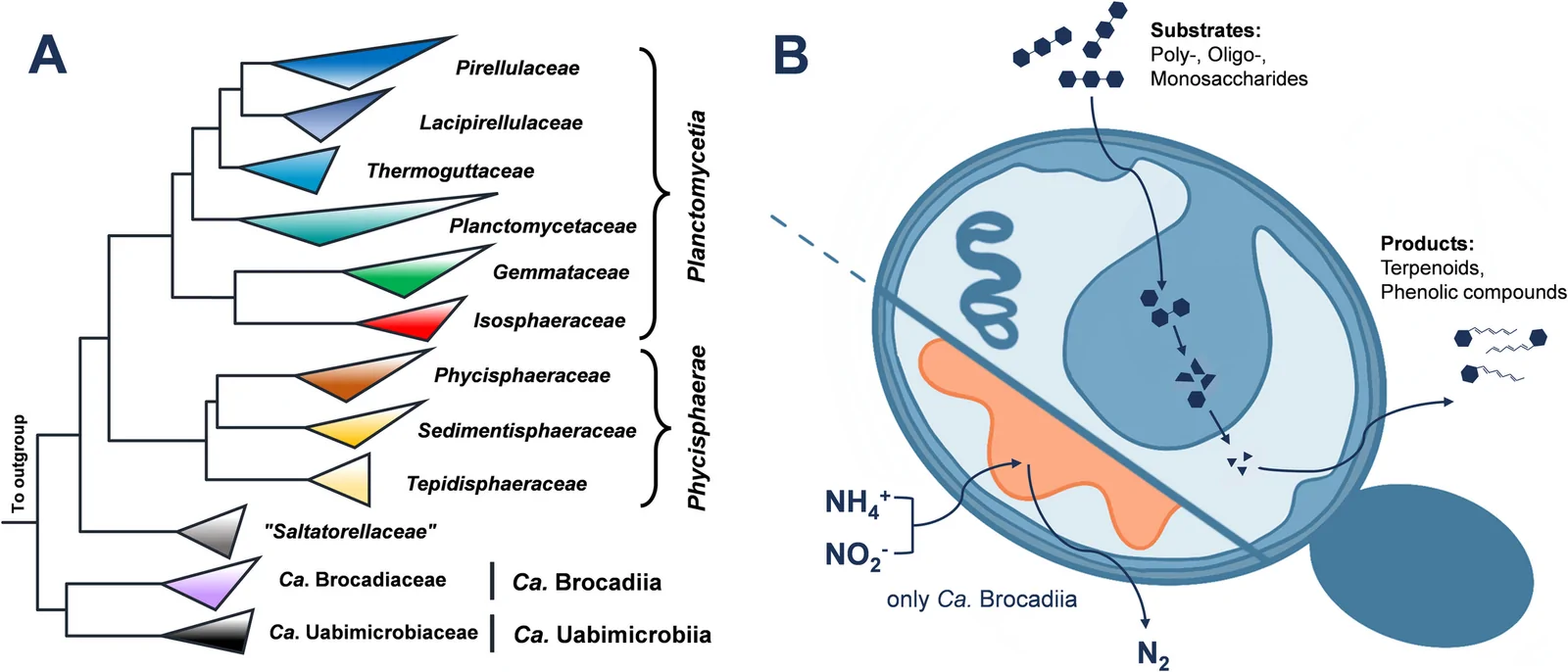
Phylogenetic tree and illustration of a planctomycetal cell. **A** 16S rRNA gene sequence-based phylogenetic tree highlighting the different families constituting the current phylum *Planctomycetota*. Families with only a single described species are not shown. The outgroup consists of sequences from members of the sister phylum *Verrucomicrobiota*. **B** Illustration of a budding planctomycetal cell. Emerging daughter cell (dark blue), cytoplasm (light blue), enlarged periplasmic space (mid blue), anammoxosome (orange)

Members of the phylum *Planctomycetota* have attracted increasing attention due to their unique cellular organization, diverse lifestyles, and metabolic capabilities (Rivas-Marín and Devos 2018; Wagner and Horn 2006; Wiegand et al. 2018). Initially described in the early twentieth century (Gimesi 1924), the phylum remained poorly understood for decades, with only a handful of species successfully isolated, cultivated and characterized (Fuerst 1995; Schlesner 1986, 1989; Schlesner and Stackebrandt 1986). However, advances in cultivation techniques and global sampling efforts over the past two decades have significantly contributed to expand the current open collection of genomes and axenic cultures (Bondoso et al. 2015; Dedysh et al. 2020; Devos et al. 2020; Kallscheuer et al. 2024; Wiegand et al. 2020). With over 100 new species described in the mentioned time span, the current phylum is taxonomically constituted by two validly described and two provisional (*Candidatus*) classes: *Planctomycetia*, *Phycisphaerae*, *Candidatus* Brocadiia, and *Candidatus* Uabimicrobiia, each with their own characteristic peculiarities (Fukunaga et al. 2009; Lodha et al. 2021; Vitorino and Lage 2022) (Fig. 1A). Central cell biological processes including peptidoglycan biosynthesis and asymmetric cell division in a budding-like process in the absence of otherwise essential bacterial proteins are not understood (Boedeker et al. 2017; Jeske et al. 2015; Rivas-Marin et al. 2023; van Teeseling et al. 2015). The same is true for the so-far unseen phagocytosis-like uptake of prey bacteria in the class *Ca*. Uabimicrobiia that might be of evolutionary significance (Shiratori et al. 2019; Wurzbacher et al. 2024). Among the described classes *Planctomycetia* is today the most extensively studied, exhibiting distinctive features such as large genomes and high numbers of genes with an unknown function (Vitorino and Lage 2022).

The phylum is increasingly recognized for the degradation of high-molecular weight carbon sources and synthesis pathways for natural products (Fig. 1B). In this article, we summarize combinatorial research advances based on bioinformatical prediction algorithms and empirical wet-lab studies focusing on the investigation of metabolic capabilities and existing and future biotechnological applications of phylum members. The current transition phase from the mere description of novel isolates to a detailed investigation of physiological principles is tightly bound to developed molecular biological tools for the construction of genetically engineered strains, covering both, gene inactivation and the introduction of heterologous genes (Jogler et al. 2011; Rivas-Marín et al. 2016).

## Substrate utilization

### Degradation of complex polysaccharides

**Fig. 2:**
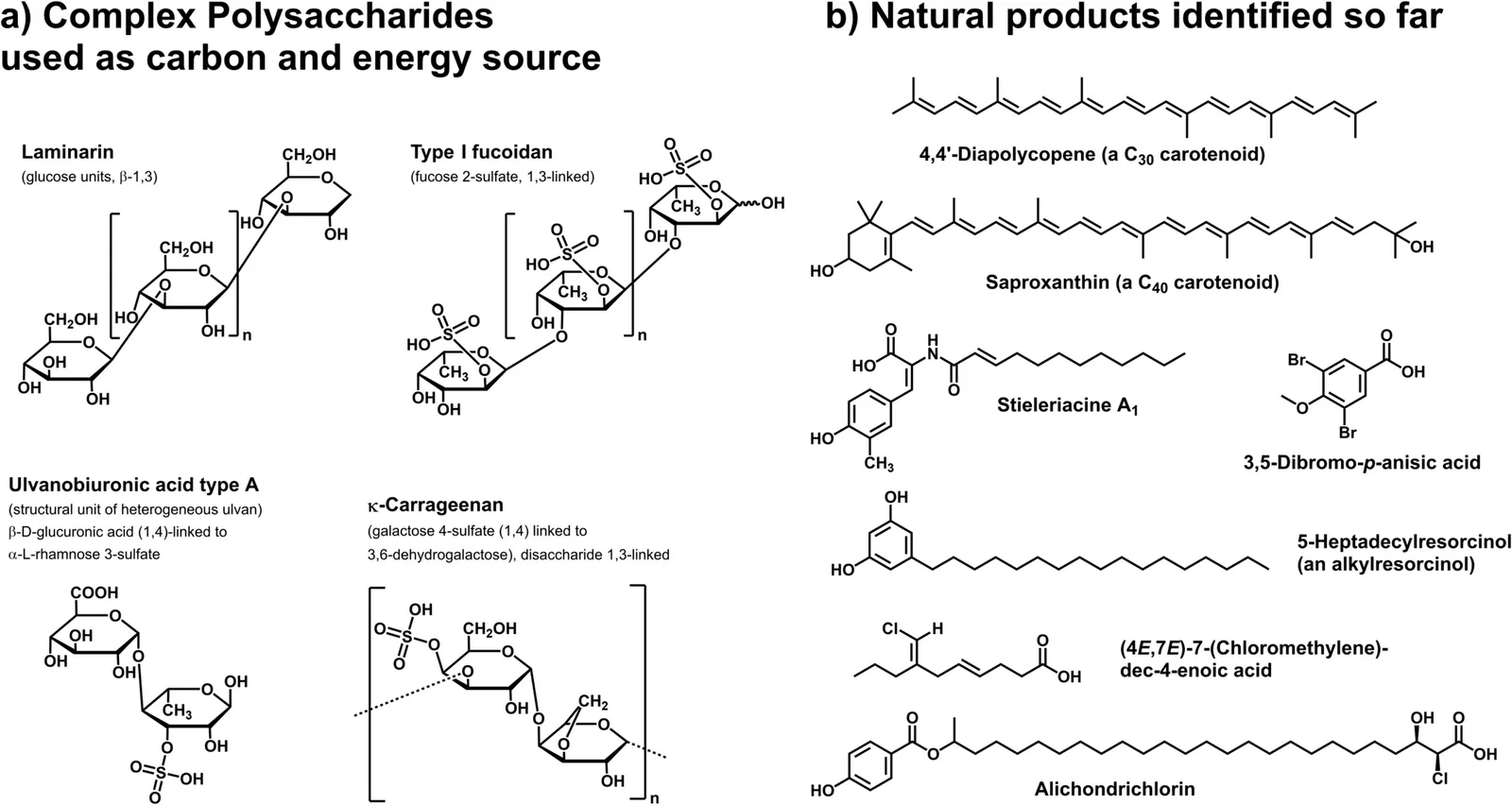
Structures of exemplary phototroph-derived polysaccharides and secondary metabolites identified in members of the phylum *Planctomycetota*. For the polysaccharides a representative unit of the molecule is shown along with the name of the monomers. The secondary metabolite structures are not shown in the chronological order of identification but were grouped based on compound class (see text for details). For compound classes with more than one identified compound (carotenoids, stieleriacines, alkylresorcinols) only the structure of the most abundant compound is depicted

Members of the phylum *Planctomycetota* occur in various aquatic and terrestrial ecosystems, with typical relative abundances of 2–13% of the bacterial community as determined in cultivation-independent amplicon sequencing studies (Ivanova et al. 2016; Kallscheuer et al. 2021; Storesund et al. 2020). However, they can be highly abundant (up to 80% relative abundance) in bacterial communities on surfaces of macroscopic phototrophs, e.g., kelp and seagrasses (Kohn et al. 2020; Lage and Bondoso 2014; Wiegand et al. 2018). This dominance is probably related to (beneficial) mutualistic interactions that include the degradation of complex and chemically modified (“decorated”) polysaccharides produced by phototrophs, e.g., laminarin, fucoidan, ulvan, and carrageenan (Fig. 2A). The degradation of polysaccharides by planctomycetes is currently investigated by three approaches: (1) morphology/structure-based approaches investigating the uptake mechanism; also taking the uncommon planctomycetal cell plan into account (Fig. 1B); (2) computational approaches by mining genomes for carbohydrate-active enzyme-encoding genes (CAZymes) and predicting substrate utilization patterns; (3) protein-biochemical approaches for investigating enzymatic functions after heterologous expression of planctomycetal genes in foreign host bacteria. Approach 1 was followed in the model species *Planctopirus limnophila* and *Gemmata obscuriglobus* (Boedeker et al. 2017). It was guided by the hypothesis that polysaccharides are taken up by cell surface-anchored pili formed by dedicated pilin-like proteins. These might serve as molecular “fishing rods” binding entire polysaccharide molecules and pulling them into the enlarged periplasmic space observed in planctomycetal cells. The first indications for such an uptake mechanism are supported by the observed internalization of gold-labelled dextran that was fed as model substrate (Boedeker et al. 2017). Approach 2 was, e.g., followed with four members of the family *Isosphaeraceae* (class *Planctomycetia*) (Ivanova et al. 2017) and recently in a broader study including genomes from all characterized members of the phylum and metagenomes (Klimek et al. 2024). The latter study also considered differences in the genome size and environmental origin of the samples. The cultivation of the model species *Rhodopirellula baltica* (“*Pirellula* sp. strain 1”, the first planctomycetal species with a sequenced genome) (Glöckner et al. 2003) has confirmed its ability to degrade sulfated polysaccharides, such as chondroitin sulfate, λ-carrageenan and fucoidan, and a diversity of putative sulfatase-encoding genes was found after inspection of the annotated genome (Wegner et al. 2013). In approach 3, a κ-carrageenase from the closely related species *Rhodopirellula sallentina* was characterized after expression of the encoding gene in *Escherichia coli* (Zhang et al. 2022b). A similar approach was followed for an exo-laminarinase from the deep-sea isolate *Stieleria* sp. TBK1r (Li et al. 2024). The strain was part of a recent computational study comparing deep sea and surface planctomycetes (Øvreås et al. 2024). The breakdown of recalcitrant fucoidan was investigated in strain HD01, another uncharacterized member of the genus *Stieleria* (Gao et al. 2024). During cultivation of the strain with fucoidan, no extracellular monosaccharides were found to be produced, which provides additional hints on the degradation of internalized polysaccharides in the periplasmic space instead of an extracellular degradation by secreted enzymes. A very recent study even suggests different strategies for fucoidan breakdown performed by different marine planctomycetes and identified the underlying gene clusters and enzyme subfamilies involved in the degradation (Pérez-Cruz et al. 2024). The resulting fucose monomers are degraded to lactate and 1,2-propanediol by a pathway that has been characterized in *P. limnophila* (former name: *Planctomyces limnophilus*) (Erbilgin et al. 2014). Part of the degradation takes place in a bacterial microcompartment for which the structural proteins are encoded in an operon along with the catabolic enzymes.

### Anaerobic ammonium oxidation and technical application of the process

The phylum also includes slow-growing autotrophic members that perform anaerobic ammonium oxidation (anammox). The existence of such bacteria has been predicted in 1977 by claims on two missing lithotrophs in nature (Broda 1977). In 1995, the first description of an anammox-performing bacterium followed (Mulder et al. 1995). It was later shown to fall within the phylum *Planctomycetota* (*Planctomycetes* at that time) (Strous et al. 1999) but failed to grow in axenic culture (Kuenen 2008; Op den Camp et al. 2007). Only enrichment cultures could be established to this very day. The identification of the monophyletic group of anammox-performing planctomycetes, now referred to as class *Ca.* Brocadiia (Fig. 1A), constituted a significant advance in the comprehension of the nitrogen cycle and explained the turnover of up to 50% of nitrogen from marine ecosystems (Arrigo 2005; Francis et al. 2007; Kartal et al. 2010; Kuenen 2008). The anammox process describes the anaerobic oxidation of ammonium and reduction of nitrite to dinitrogen gas with the toxic intermediate hydrazine (Strous et al. 1998) and takes place in a dedicated compartment in these specialized planctomycetes, the anammoxosome (Jogler 2014; van Niftrik et al. 2004) (Fig. 1B). Biotechnological application of this process during nitrogen elimination in wastewater was evident, but the anammox process needed to be stabilized. Denitrifying fluidized bed reactors, sequencing batch reactors, membrane bioreactors and (semi-)continuous stirred tank reactors have been established for cultivation and are continuously optimized (Ding et al. 2018; Güven et al. 2004; Mulder et al. 1995; Strous et al. 1998; Wang et al. 2009). By adding nitrite instead of nitrate (previously thought to be the contributing compound), the unstable process could be stabilized (Kuenen 2008; Op den Camp et al. 2007). Several studies have emphasized that the anammox process is sensitive to changes in substrate concentration (Chang et al. 2023; Chen et al. 2023; Chi et al. 2021), temperature (Byrne et al. 2008; Sobotka et al. 2021; Weralupitiya et al. 2021), pH (Li et al. 2020; Ma et al. 2022), salinity (Weralupitiya et al. 2021; Zhang et al. 2022a), presence of organic compounds like antibiotics (Gamoń et al. 2022; Lotti et al. 2012), and heavy metals (Lotti et al. 2012). The coupling of different nitrogen removal processes turned out to be advantageous. To this end, one-stage and two-stage processes have been developed; the former generally being more cost-effective (Wyffels et al. 2004) and the latter being more stable in case of changing biogeochemical parameters (Shalini and Joseph 2012; Van Dongen et al. 2001; Vázquez-Padín et al. 2009). Examples of one-stage processes include *Simultaneous partial Nitritation, Anammox, Denitrification, and COD* [chemical oxygen demand] *Oxidation* (SNADCO)/S*imultaneous Carbon Oxidation, partial Nitritation, Denitritation and Anammox* (SCONDA) and *Simultaneous Partial Nitritation, Anammox and Denitrification* (SPNAD) that can lead to nitrogen removal efficiencies of up to 81% and 94–99%, respectively (Guo et al. 2020; Liu et al. 2021; Zhang et al. 2019; Zhou et al. 2018). Technical challenges remain mostly because of physiological restraints of anammox-performing planctomycetes, for example slow growth with minimal generation times in the range of days to weeks and sensitivity to changing biogeochemical conditions (Guo et al. 2020; Wang et al. 2022; Wu et al. 2022).

## Exploring the planctomycetal secondary metabolite portfolio

### Antimicrobial activity and predicted biosynthetic gene clusters

In the 2010s, more genomes and axenic cultures of planctomycetes became publicly available and the phylum was soon after recognized as an untapped source for the discovery of novel secondary metabolites with potential health-promoting bioactive properties (Jeske et al. 2013).

After the first reports on antimicrobial and anti-cancer activities in extracts of planctomycete cultures (Calisto et al. 2019; Graça et al. 2016), research has focused on both, the automated prediction of biosynthetic gene clusters (BGCs) and untargeted bioprospection studies yielding novel molecule structures. Genome mining of approx. 100 characterized strains showed that many of the predicted BGCs do not cluster with BGCs involved in the formation of known compounds in well-investigated bacterial “talented producers” (Wiegand et al. 2020). This not only suggested an uncharacterized secondary metabolite portfolio of the phylum, but also a limited predictive power of the algorithms that have been developed based on data of well-investigated clusters in distantly related taxa. In other words, many of the planctomycetal BGCs might have escaped computational analyses so far. The set of planctomycetal BGCs is regularly updated by in silico analyses including more recent isolates (Calisto et al. 2025; Kallscheuer and Jogler 2021). However, most clusters remain unlinked to actual biosynthetic pathways or molecule structures. Most of the compounds have been identified by untargeted cultivation and extraction approaches and only in some cases could be linked to a putative BGC. Effective species description articles published in the last 5 years regularly reported on antimicrobial activities in novel isolates belonging to different families, substantiating the assumed role of planctomycetes as untapped source of bioactive compounds (Belova et al. 2020; Gao et al. 2024; Kumar et al. 2024; Vitorino et al. 2022a, 2022b).

### Characterized planctomycetal secondary metabolites

Approximately half of the currently described members of the phylum are pigmented. Colors range from red to pink/salmon or in rare cases orange and could be traced back to the formation of carotenoids. The limnic model planctomycete *P. limnophila* (family *Planctomycetaceae*) was shown to produce C_30_ carotenoids via the triterpene precursor squalene, whereas C_40_ and C_45_ carotenoids have been identified in two marine strains of the family *Pirellulaceae* (Kallscheuer et al. 2019; Santana-Molina et al. 2022). The light-dependent regulation of carotenoid biosynthesis was recently analyzed in strains belonging to the family *Isosphaeraceae* (Ivanova et al. 2025). The C_40_ carotenoid-forming type strain of the marine species *Rhodopirellula rubra* (family *Pirellulaceae*) was used as supplementary food source for the water flea *Daphnia magna*. Although the observed positive effects were not exclusively traced back to anti-oxidative properties of carotenoids, the study suggests applications of planctomycetes in aquafarming (da Conceição et al. 2019). Additional natural compounds produced by phylum members (Fig. 2B) function as potential chemical mediators during the interaction of planctomycetes with phototrophs or other heterotrophic bacteria. Stieleriacines, a class of *N*-acylated tyrosine derivatives, have been identified in two members of the genus *Stieleria* (family *Pirellulaceae*) (Kallscheuer et al. 2020; Sandargo et al. 2020). The supplementation of stieleriacine A_1_ promoted the biofilm formation of the marine alphaproteobacterium *Phaeobacter inhibens*, but reduced biofilm formation of a different species, suggesting a role in the alteration of the species composition in surface-associated bacterial communities (Kallscheuer et al. 2020). *N*-acyl tyrosines and other *N*-acylated amino acids have been functionally linked to the formation of extracellular polymeric substances (Craig et al. 2011) and are promising sustainable alternatives to currently used surfactants (Haeger et al. 2024).

To date, the only targeted approach based on bioinformatically predicted BGCs focused on a three-gene BGC that includes a putative type III polyketide synthase-encoding gene (Milke et al. 2024). Genes from six planctomycetes with different variations of the cluster have been heterologously expressed in an engineered strain of *Corynebacterium glutamicum* that was previously engineered towards the production of plant-derived polyphenols (Kallscheuer et al. 2016). It has been shown that the investigated BGC is involved in the synthesis of alkylresorcinols of so far unclear function. Comparative analyses of the type III polyketide synthase gene sequence point towards horizontal gene transfer of the BGC between planctomycetes and picocyanobacteria which provides additional hints on frequent natural interaction. The picocyanobacterial counterpart cluster was shown to produce similar alkylresorcinols designated hierridins (Costa et al. 2019).

An uncharacterized member of the phylum, strain 10988, produces the dibrominated phenolic compound 3,5-dibromo-*p*-anisic acid that likely functions as a plant toxin (Panter et al. 2019). The compound may promote the decay of the phototroph at the end of algal blooms that will benefit planctomycetes and other heterotrophic bacteria that can degrade polysaccharides in the algal biomass. Halogenated compounds, in this case a chlorinated fatty acid, were already reported in *R. baltica* (family *Pirellulaceae*) back in 2011 (Lee et al. 2010). Recently, alichondrichlorin, an ester of 4-hydroxybenzoic acid and a chlorinated fatty acid, was identified in *Alienimonas chondri* (family *Planctomycetaceae*) (Vitorino et al. 2025). The compound showed anti-tumour activity. Cabrillospirals identified in a Small Molecule In Situ Resin Capture approach have been loosely linked to a BGC in a planctomycetal metagenome-assembled genome as the best candidate (Bogdanov et al. 2024). Taken together, five compounds/compound classes have been reported to be produced by planctomycetes, all by members of the class *Planctomycetia* (Fig. 2B). Carotenoid and alkylresorcinol biosynthesis have been linked to genes or clusters (Milke et al. 2024; Santana-Molina et al. 2022), whereas pathways and/or candidate genes have been postulated for biosynthesis of stieleriacines, 3,5-dibromo-*p*-anisic acid and cabrillospirals (Bogdanov et al. 2024; Kallscheuer et al. 2020; Panter et al. 2019). Genes coding for enzymes involved in alichondrichlorin biosynthesis are currently unknown.

## Development of tools for the genetic manipulation of planctomycetes and outlook

The current lines of research on planctomycetes include the investigation of cell biological principles, such as cell division and peptidoglycan biosynthesis, carbon source uptake and degradation as well as natural compound biosynthesis. All of them benefit from genetic tools for the construction of gene inactivation mutants and introduction of heterologous DNA into the chromosome. Basic tools for an untargeted transposon-driven or targeted homologous recombination-based genome modification have been developed for five members of the phylum (Jogler et al. 2011; Rivas-Marín et al. 2016) (families *Pirellulaceae*, *Planctomycetaceae* and *Gemmataceae*). Perspectives and current limitations during genetic engineering have been summarized (Kallscheuer and Jogler 2021). While gene deletion mutants have become an integral part of research studies with the previously tested strains of the class *Planctomycetia* (Milke et al. 2024; Rivas-Marin et al. 2023; Santana-Molina et al. 2022), members of the other classes (*Phycisphaerae*, *Ca*. Uabimicrobiia and *Ca*. Brocadiia) are more challenging to handle and have not yet been tested for genetic accessibility. This is due to several reasons including resistance to commonly used antibiotics, lack of axenic cultures, growth in macroscopic aggregates or lack of growth on plates.

Recently, the genetic engineering toolbox for the limnic model strain *P. limnophila* has been extended by testing additional fluorescent reporter proteins and the introduction of an inducible gene expression system based on a native rhamnose-responsive regulatory circuit (Haufschild et al. 2025). Ongoing efforts focus on the construction of a replicative plasmid based on broad-host range replicons for Gram-negative bacteria and elements from native plasmids predominantly from members of the *Isosphaeraceae* family (Quiñonero-Coronel et al. 2024). While additional sets of genetic elements active in planctomycetes are currently explored, optimization potential on the level of cultivation is exploited to accelerate growth and increase biomass formation (Kruppa and Czermak 2022; Kruppa et al. 2021).

The phylum *Planctomycetota* is among the known bacterial phyla with the highest relative number of genes with unknown function (Overmann et al. 2017). This may be due to the limited predictive power of gene annotation or genome mining algorithms (e.g., antiSMASH) trained with datasets of well-characterized phyla but is also the result of the presence of several planctomycete-specific protein domains of unknown function (DUFs). In this light, wet lab research needs to be prioritized over in silico analyses of genome-encoded features also beyond the description of novel isolates. Research articles published in the last 5 years are good indicators for the upcoming exploitation of the biotechnological potential of phylum members. Future research will surely yield bioactive compounds of commercial value, but will also motivate to explore other little-studied or yet non-cultivable bacteria, the “microbial dark matter.”

## Data Availability

No datasets were generated or analysed during the current study.
